# Survival analysis of conservative vs. dialysis treatment of elderly patients with CKD stage 5

**DOI:** 10.1371/journal.pone.0181345

**Published:** 2017-07-24

**Authors:** Roman Reindl-Schwaighofer, Alexander Kainz, Michael Kammer, Alexandra Dumfarth, Rainer Oberbauer

**Affiliations:** 1 Department of Nephrology, Krankenhaus der Elisabethinen, Linz, Austria; 2 Department of Nephrology, Medical University of Vienna, Vienna, Austria; 3 Center for Medical Statistics, Informatics and Intelligent Systems, Medical University of Vienna, Vienna, Austria; Postgraduate Medical Institute, INDIA

## Abstract

Elderly patients represent a growing population among people suffering from ESRD. So far only limited data on actual survival benefits of elderly adults initiating dialysis have been published. Besides the high burden of preexisting comorbidities, dialysis treatment itself may be associated with a further deterioration in functional status in this population. We retrospectively analyzed the Austrian Dialysis and Transplant Registry and identified 8,622 patients who started maintenance hemodialysis after the age of 65 years between 2002 and 2009. We compared this data set to a cohort of 174 patients aged over 65 years with CKD stage 5 who progressed to an eGFR < 10ml/min/ and were managed conservatively in the same era. All patients who died of malignant disease were excluded from this analysis. The risk of mortality was analyzed using multivariable Cox proportional hazards models. Furthermore, a parametric model of time to event analysis was used for visualization of changing risk over time and precise calculation of time to equal risk assuming a Weibull distribution. Hemodialysis treatment was associated with a decreased risk for death with a HR of 0.23 (95% CI 0.18 to 0.29; p<0.001) compared to conservative treatment. The time to event analysis however showed, that although survival was initially superior in the hemodialysis group, hazards crossed thereafter. Time to equal risk was 2.9 months and 1.9 months for female and male patient aged 65, respectively, and decreased to one month in the very elderly aged 95. Elderly patients with ERSD did benefit from initiation of hemodialysis, as the conservative group showed a very high initial mortality rate. This survival benefit of dialysis treatment however did not persist beyond the first two months compared to survivors of the conservative group.

## Introduction

Over the last decades, end stage renal disease (ESRD) patients became significantly older and sicker [1]. Today almost every other dialysis patient in industrialized countries is older than 65 years and patients older than 75 years represent the fastest growing age group of prevalent dialysis patients [2]. This development is mainly driven by the continuing increase in life expectancy in these countries and a more liberal access to renal replacement therapy for older patients [3]. In the United States almost 7% of the total Medicare and non-Medicare healthcare budget is spent on the care of ESRD patients while the prevalence is only 1.3% of all Medicare beneficiaries [4].

Epidemiological and survival data of dialysis treatment and kidney transplantation are well documented through country specific registries such as the European Dialysis and Transplant Association (EDTA) or the Austrian Dialysis and Transplant registry (OEDTR). For conservatively managed patients no such data repositories exist [5]. Dialysis is a life-saving procedure for patients with ESRD. However, it is associated with considerable comorbidities and a high mortality that is driven by frequent infections, chronic inflammation, accelerated atherosclerosis and malnutrition. In patients with severe comorbid illnesses including cardiovascular disease and diabetes the short-term mortality is often very high despite the initiation of renal replacement therapy [6–8]. Additionally, initiation of hemodialysis in elderly patients may further deteriorate functional status and reduce quality of life [9–11]. Transportation to and from hemodialysis units is often an all-day affair for patients with limited mobility, leaving very little time for other activities on hemodialysis days including reduced nutritional intake [12].

When evaluating elderly patients for renal replacement therapy (RRT) several additional parameters have to be taken into consideration including the remaining live expectancy of the patient on dialysis which is most often limited by comorbidities including diabetes and advanced vascular disease. In elderly patients or younger patients in a palliative setting ESRD may adequately be managed conservatively without the initiation of RRT especially in individuals with relatively physical wellbeing in terms of ESRD related symptoms [12]. This includes correction of acid base and electrolyte disorders, fluid and blood pressure control and anemia management. It has been shown that hemodialysis itself leads to a progressive loss of kidney function [13]. However, conservative management can only be continued as long as clinical symptoms of uremia are controlled by supportive medical treatment.

## Materials and methods

This retrospective analysis has been approved by the ethics committee of the Medical University of Vienna. We only received deidentified data from the transplant registry. Data obtained from the medical records of the outpatient department was deidentified following identification of subjects that met the inclusion criteria. Only the first author had access to identifying data as part of the initial data mining process before deidentification of the data set (that was performed by the first author). The IRB consented to a retrospective data analysis without need for an individual consent. Data evaluation and deidentification was performed according to the IRB requirements.

In this observational analysis we searched the Austrian Dialysis and transplant registry (OEDTR) and identified 8.622 incident hemodialysis patients aged 65 years and older who started chronic renal replacement therapy from January 2002 until December 2009.

Subsequently we searched the medical records of a tertiary care hospital in Linz, Austria, for all patients with CKD stage 5 aged > 65 years that were managed conservatively in the same era. We included all patients with a reported eGFR < 10ml/min/1,73m^2^. We then excluded all patients who died due to a malignant disease and all patients whose renal function recovered within three months (defined as an eGFR above 15ml/min/1.73 m^2^) to account for episodes of reversible kidney injury. We thereby identified a cohort of 174 conservatively managed patients with CKD stage 5 aged 65 and older.

In order to adjust for the different numbers of patients between the groups, we calculated a propensity score by applying bootstrap resampling with 1000 runs including all patients of the conservative group and the same number of randomly chosen patients from the dialysis group. The propensity score included comorbidities (COPD, diabetes, hypertension, heart disease, neoplasia, liver disease and vascular disease) as well as age and sex.

Demographic variables were compared by the two-sample t-test. For categorical variables the chi-square test was applied. Kaplan-Meier (KM) plots were used to visualize the association of dialysis treatment with patient mortality. Significance was calculated by log-rank test. A multivariable Cox model was computed, which included modality of treatment, age, sex and all comorbidities as covariates. To investigate the change of the hazards over time Cox models including the same covariates were computed for two sequential time periods (0–2 months since study inclusion and after 2 months since study inclusion). Administrative censoring at 2 months was applied for the first model. Only patients who survived the initial period entered the model for the subsequent period.

Furthermore, we calculated a parametric model (assuming a Weibull distribution) for survival analysis stratifying by treatment modality (dialysis vs. conservative) to assess time to equal risk. Starting point for all further survival analysis was the beginning of dialysis treatment or the first GFR below 10 ml/min/1,73m^2^. All eGFR calculations are based on the CKD-EPI formula. A p-value less than 0.05 was considered statistically significant. For all analyses SAS for Windows 9.2 (The SAS Institute, Inc., Cary, North Carolina, USA) was used.

## Results

The demographic data for both groups are presented in Table 1. Patients in the conservative group were significantly older with a mean age of 81 years compared to 74 years in the dialysis group (p<0.001). Both groups also significantly differed by gender. Male patients represented the majority in the dialysis group with 54% compared to only 5% in the conservative group (p<0.001). Patients in the conservative group had significantly more comorbidities. Demographic variables that were unequally distributed between the groups include COPD, heart disease, liver disease, and neoplasia (p<0.001)

**Table 1:** Demographics. Values are presented as mean (standard deviation) or as percentage.

	Hemodialysis (n = 8,622)	Conservative treatment (n = 174)	p-value
Age (years)	74.06 (5.78)	81.22 (7.23)	<0.001
Sex (f / m)	46% / 54%	95% / 5%*	<0.001
COPD (no / yes)	91% / 9%	82% / 18%	<0.001
Diabetes mellitus (no / yes)	63% / 37%	66% / 34%	0.39
Heart disease (no / yes)	42% / 58%	30% / 70%	<0.001
Hypertension (no / yes)	37% / 63%	40% / 60%	0.341
Liver disease (no / yes)	94% / 6%	87% / 13%	<0.001
Neoplasia (no / yes)	88% / 12%	84% / 16%	<0.001
Vascular disease (no / yes)	54% / 46%	49% / 51%	0.2

**Fig 1:**
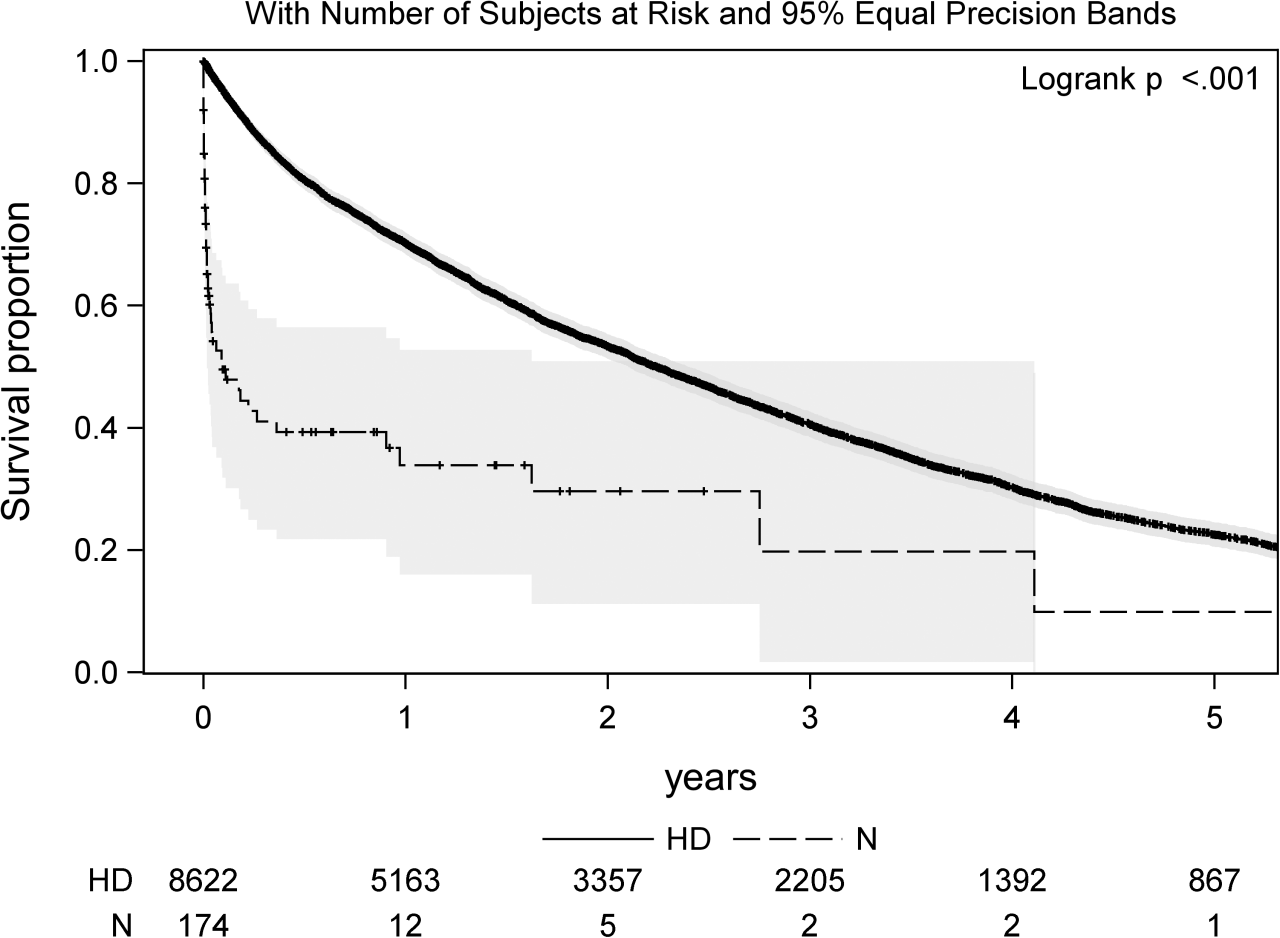
Patient survival stratified by dialysis treatment (HD) vs. conservative management (N) including 95% equal precisions bands.

Fig 1 shows a Kaplan-Meier (KM) plot to visualize patient mortality stratified by treatment modality. Median survival time was 26.9 months (95% CI 25.8 to 28.0 months) and 1.1 months (95% CI 0.4 to 10.8 months) in the dialysis and conservative group, respectively. Median survival in the hemodialysis group decreased from 30.2 months (CI 95% 28.7 to 31.6) in patients aged 65 to 75 to 21.8 (CI 95% 20.1 to 24.1), 19.6 (CI 95% 17.8 to 22.2) and 17.7 months (CI 95% 13.8 to 20.8) in patients aged 75–80, 80–85 and > 85 years, respectively. * The female majority arose from the predominant female patient population of this medical hospital.

Hemodialysis treatment was associated with a survival benefit with a HR for death of 0.39 (95% CI 0.32 to 0.47; p<0.001). Age and comorbidities such as diabetes mellitus, heart disease, liver disease and vascular disease were all associated with an increased mortality, whereas hypertension had a beneficial effect on survival in our analysis (Table 2). To further assess changing hazards over time we calculated hazard ratios for two time periods. In the initial two months the HR for death comparing dialysis treatment vs. conservative managed was 0,07 (95% CI 0.06–0.1). For the subsequent period this survival benefit did not persist. There was a non-significant trend to better survival in the conservatively managed group with a HR of 1.14 (95% CI 0.61–2.13).

**Table 2:** Hazard ratios (HR) and confidence intervals (CI) for death based on the multivariable Cox proportional hazards model.

	Overall HR (95 % CI; p-value)	HR for 0–2 months (95 % CI; p -value)	HR after 2 months (95 % CI; p -value)
Hemodialysis vs. no dialysis	0.23 (0.18–0.29, p<0.001)	0.07 (0.06–0.1, p<0.001)	1.14 (0.61–2.13, p = 0.67)
Age (decade)	1.42 (1.36–1.49, p<0.001)	1.45 (1.29–1.64, p<0.001)	1.41 (1.34–1.48, p<0.001)
Sex (f vs. m)	0.92 (0.86–0.98, p = 0.01)	1.06 (0.91–1.23, p = 0.48)	0.93 (0.88–0.99, p = 0.02)
COPD (no vs. yes)	0.86 (0.77–0.95, p = 0.003	0.77 (0.58–1.02, p = 0.06)	0.89 (0.8–0.99, p = 0.03)
Diabetes mellitus (no vs. yes)	1.20 (1.13–1.27, p<0.001)	1.12 (0.94–1.33, p = 0.19)	1,23 (1.15–1.31, p<0.001)
Heart disease (no vs. yes)	1.1 (1.03–1.17, p = 0.01)	1.55 (1.29–1.86, p<0.001)	1.03 (0.96–1.1, p = 0.44)
Hypertension (no vs. yes)	0.57 (0.54–0.61, p<0.001)	0.4 (0.34–0.47, p<0.001)	0.6 (0.56–0.63, p<0.001)
Liver disease (no vs. yes)	1.12 (1.0–1.26, p = 0.05)	1.5 (1.15–1.96, p = 0.001)	1.16 (1.02–1.31, p = 0.02)
Neoplasia (no vs. yes)	1.05 (0.97–1.15, p = 0.24)	1.02 (0.82–1.28, p = 0.84)	1.16 (1.02–1.31, p = 0.02)
Vascular disease (no vs. yes)	1.11 (1.04–1.18, p = 0.002)	0.97 (0.81–1.15, p = 0.69)	1.13 (1.06–1.21, p<0.001)

**Fig 2:**
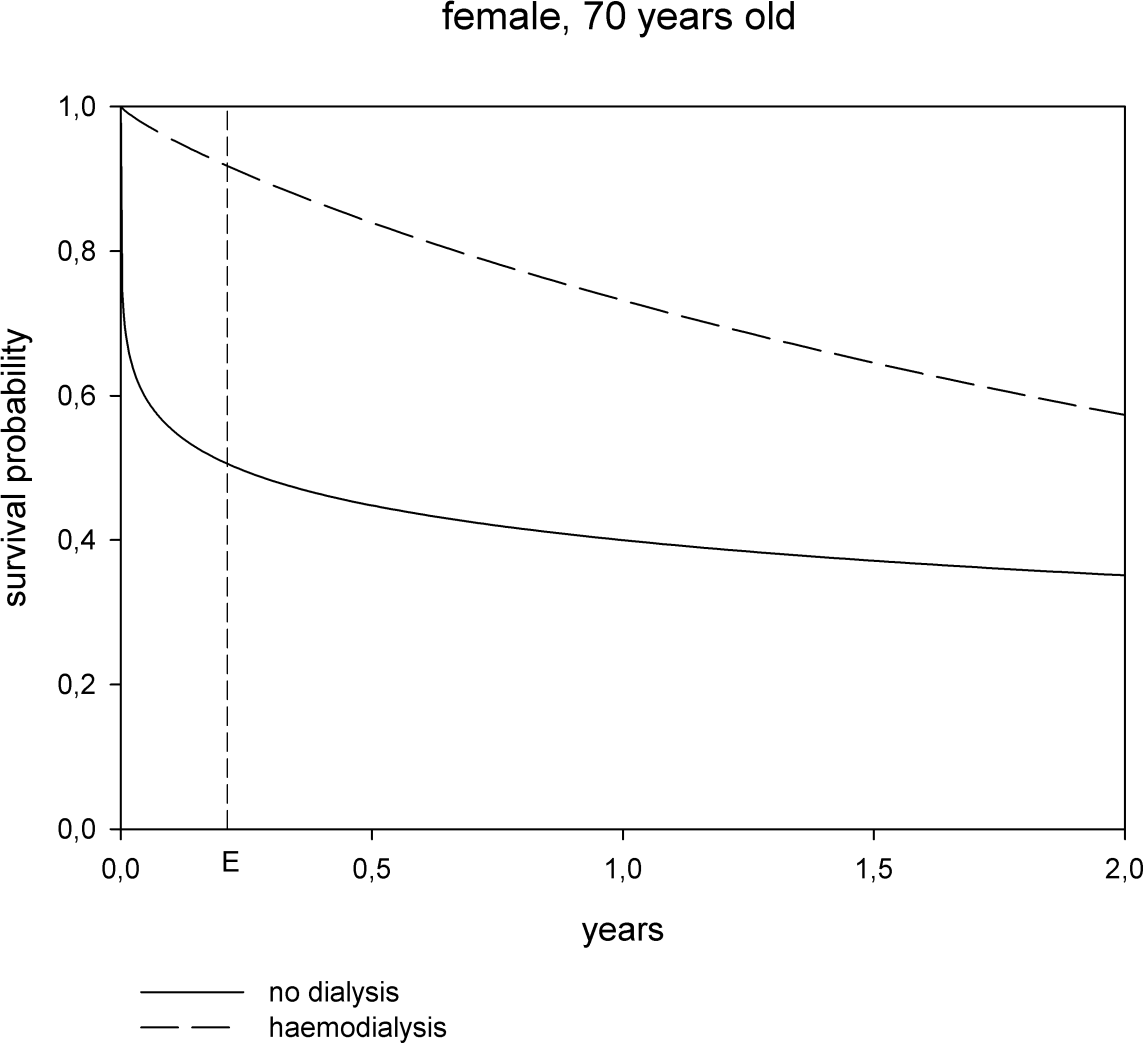
Survival probability modeled by Weibull distribution and stratified by treatment. Dialysis treatment vs. conservative management for a female patient aged 70 years. *E* marks the time to equal risk (the slopes of the two survival curves are the same).

**Fig 3:**
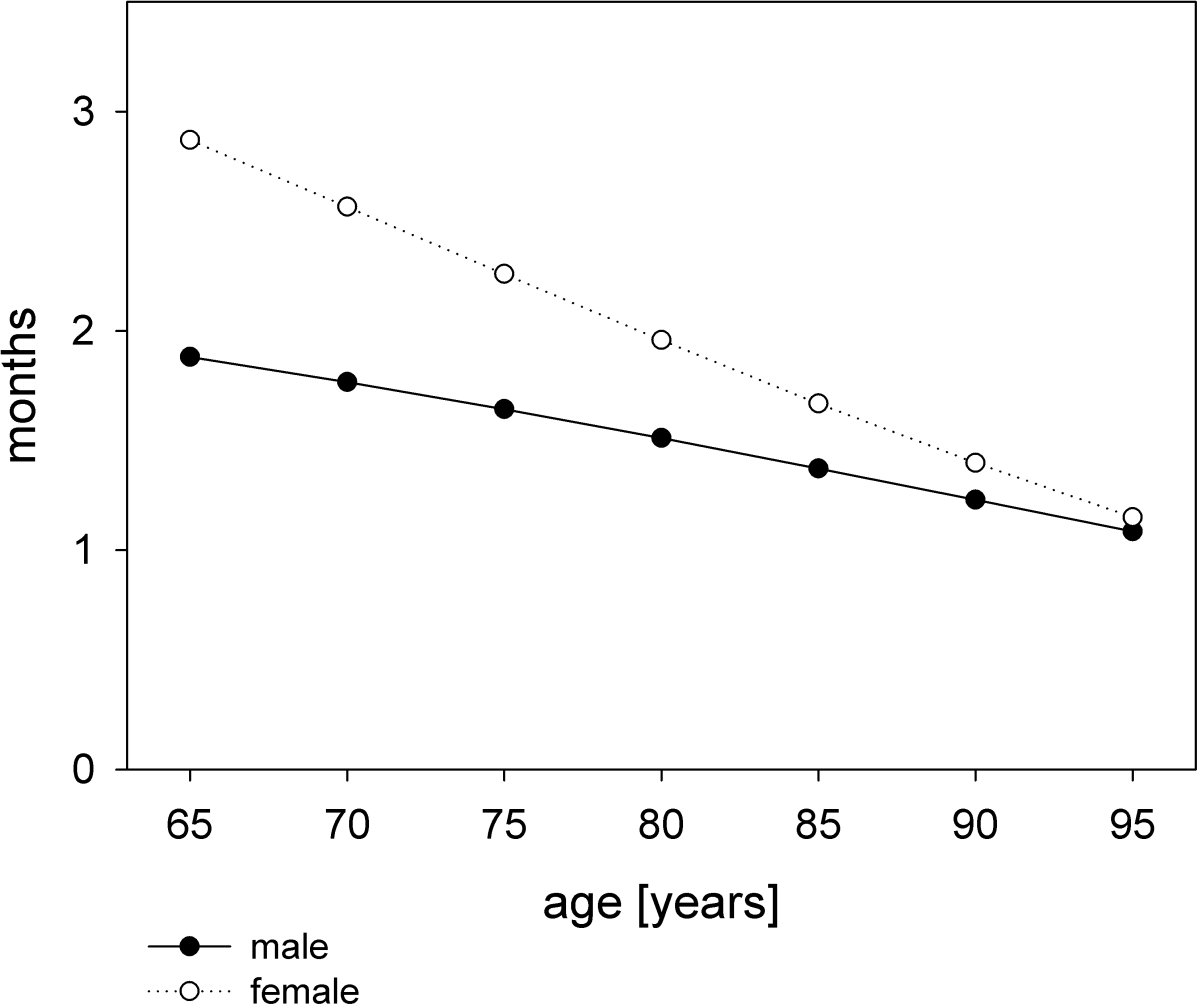
Months to equal risk compared between dialysis treatment and conservative management.

The parametric analysis showed, that although survival was initially superior in patients treated with hemodialysis, hazards crossed thereafter (Fig 2). Time to equal risk was calculated at 2.9 months for a female patient aged 65 years and decreased to 1 month at 95 years. For male patients time to equal risk decreased from 1.9 months at 65 years to one month at 95 years (Fig 3).

## Discussion

Our data shows an overall survival benefit for elderly patients started on hemodialysis treatment compared to conservative management. This hazard ratio is driven by the very high initial mortality in the conservatively managed patients with a median survival of only one month.

Our results are in line with previous published data as all studies so far showed an overall survival benefit for elderly patients treated with RRT compared to maximum conservative management alone [14–19]. However, selected individuals opting for a conservative management show an unexpected longevity [18, 20]. Besides, an increase in survival time alone may not be the main objective in the care of elderly patients with ESRD [5]. Older patients on hemodialysis have a great symptom burden (pain, fatigue, pruritus, constipation), which can only be poorly addressed by the dialysis treatment and often remain unchanged [12].

Across countries, mortality rates among hemodialysis patients aged above 65 years show a great variance. In patients older than 80 years a 1-year mortality rate of 46% was reported in a review based on the USRDS from 2007 [21]. The REIN registry data showed a 6-month mortality of 19% in dialysis patients aged 75 years or older [22]. In the DOPPS study, median survival of patients older than 75 years of age varied between 1.6 and 5.4 years [23].

Over the last decade several studies on the survival of elderly patients with ESRD opting for conservative treatment have been published. Joly et al. reported a median survival of 28.9 months in patients opting for RRT compared to 8.9 months in the conservatively managed cohort [15]. Measuring from a higher eGFR starting point of 15 ml/min, Murtagh et al. reported a median survival of 22 months in a conservatively managed cohort [16]. In a study on 202 elderly patients (>70 years) with ESRD Carson et al. reported a median survival of 13.9 months with maximum conservative treatment compared to 38.8 months in patients started on hemodialysis [18].

In the largest cohort analysis that has been published so far including 844 patients (82% on RRT, 18% with conservative management), Chandna et al. reported that median survival from entry into stage 5 chronic kidney disease was less in conservatively managed patients compared to RRT (21 months vs. 67 months) [17]. However, in patients aged >75 years with high comorbidity and diabetes this survival benefit decreased to only 4 months. Similar findings were reported by Hussain et al. showing that the survival benefit of RRT was lost in patients > 80 years with a poor performance status or high co-morbidity scores [14].

In our cohort median survival in the conservatively managed patients was very poor compared to most previously published data. In the comparative statistics, the conservative group showed a higher burden of comorbid illnesses and was significantly older. There were significantly more male patients in the dialysis group (55%) compared to only 5% in the conservatively managed group. A gender discrepancy in prevalent dialysis patients has been observed before [24]. Potential factors driving this observation are currently investigated in large registry analyses. The factors contributing to this gender discrepancy remain elusive.

The observed high mortality in the conservative group may further be driven by the data mining approach based on serum creatinine values that was applied to identify CKD patients with renal failure in a clinical database. This may result in a substantial bias by indication including severely ill patients that did not qualify for hemodialysis in the first place. We therefor addressed the selection bias by excluding all patients who died due to a malignant disease and by calculating a propensity score for treatment based on all clinical covariates that are available in the dialysis registry data. However, other important confounders such as tobacco use, BMI, nutritional status (albumin) or co-medication are not routinely recorded in the ÖDTR and were not included in the propensity score resulting in residual confounding. We are therefore aware that patients in the conservative group may have more comorbidities resulting in an indication bias that cannot be completely addressed in an observational study design. Another limitation of this analysis is the low number of patients with conservative treatment that remain in the study for longer than one year. We therefore focus on results within the first few months after study inclusion.

Comparing survival in conservatively managed patients between different cohorts (and to patients on hemodialysis) is difficult as there is no clear starting point for analysis and thus a potential lead time bias. The inclusion criteria of an eGFR <10ml/min/1,73m^2^ in the conservatively managed patients was chosen arbitrarily. No data on the actual eGFR at the initiation of dialysis was available, as no creatinine levels were recorded in the OEDTR. Carson et al. reported that dialysis is usually started at a median GFR of 10.8 ml/min in their cohort analysis on the management of ESRD in the elderly.

The right time to start on chronic renal replacement therapy in elderly patients remains elusive and recommendations of international societies have changed over the last decade [25, 26]. In the general population early initiation of renal replacement therapy (RRT) did not provide a survival benefit [27]. Therefore initiation of dialysis above a GFR of 10ml/min is only recommended in a minority of subjects with overt clinical symptoms of uremia (such as frequent emesis, uremic pericarditis or intractable hyperkalemia) and fluid overload that cannot be treated conservatively.

Additionally, eGFR alone as a continuous parameter remains a poor indicator for the timing of dialysis initiation. However, patients with an eGFR below 10 ml/min are generally considered eligible for renal replacement therapy. In our population, no information on uremic symptoms were available for both groups. Lead time bias is therefore difficult to assess and may be another source of confounding in an observational study. To address this issue we performed sensitivity analysis at a higher eGFR threshold of 15 ml/min showing similar results.

Despite an overall survival benefit in the dialysis group, a time to event analysis showed that hazards for death crossed after two to three months resulting in a better survival in conservatively managed patients who survived for more than two months. Using time to equal risk analysis allowed us to calculate the time point at which a conservatively treated patient had the same risk for death compared to a patient on hemodialysis. Time to equal risk is the time point when the slopes of the two survival curves are the same. After this time point the risk to die is higher for a dialysis patient than for one treated conservatively. Time to equal survival on the contrary is the time point, when the two survival curves cross. Time to equal risk analysis help to identify the time point when one treatment becomes superior to another when analyzing cohorts with changing hazards over time.

The time to event analysis further suggests that the benefit of dialysis treatment decreases with age. Our data raise questions on the optimal treatment of elderly patients with ESRD that cannot be addressed in an observational study.

This retrospective analysis did not include patients treated with peritoneal dialysis. Shum et al. reported that peritoneal dialysis may be suitable treatment option for elderly patients with ESRD [28].

## Conclusion

Despite the intrinsic limitations of an observational study design (lead time bias, indication bias) our data clearly showed, that in conservatively treated patients with CKD stage 5 progressing to an eGFR < 10ml/min/1,73m^2^ mortality is very high. Similar to previously published data on the management of elderly patients with CKD our data supports that dialysis treatment improves survival of elderly patients with ESRD compared to conservative management. However, this survival benefit decreases with age and selected individuals with ESRD show long term survival without renal replacement therapy. The time to event analysis further showed, that following the initially very high mortality in the conservative group, hazards crossed in patients surviving for more than three months resulting in a decreased risk of death. Further implications on treatment strategies have to be evaluated in a prospective trial.
